# Shallow kinetics induced by a metronome (SKIM): A novel contactless respiratory motion management

**DOI:** 10.1002/acm2.14147

**Published:** 2023-09-06

**Authors:** James Sohn, Mitchell Polizzi, Philip Reed McDonagh, Christopher Guy, Rabten Datsang, Elisabeth Weiss, Siyong Kim

**Affiliations:** ^1^ Department of Radiation Oncology Northwestern Memorial Hospital Northwestern University Feinberg School of Medicine Chicago Illinois USA; ^2^ Department of Radiation Oncology Virginia Commonwealth University Richmond Virginia USA

**Keywords:** fast shallow‐breathing (FSB), free breathing (FB), metronome, motion management, stereotactic body radiation therapy (SBRT)

## Abstract

**Objectives:**

As an alternative to conventional compression amidst the COVID‐19 pandemic, we developed a contactless motion management strategy. By increasing the patient's breathing rate to induce shallow breathing with the aid of a metronome, our hypothesis is that the motion magnitude of the target may be minimized without physical contact or compression.

**Methods:**

Fourteen lung stereotactic body radiation therapy (SBRT) patients treated under fast shallow‐breathing (FSB) were selected for inclusion in this retrospective study. Our proposed method is called shallow kinetics induced by a metronome (SKIM). We induce FSB by setting the beats‐per‐minute (BPM) high (typically in the range of 50–60). This corresponded to a patient breathing rate of 25–30 (breathing) cycles per minute. The magnitude of target motion in 3D under SKIM was evaluated using 4DCT datasets. Comparison with free breathing (FB) 4DCT was also made for a subset for which FB data available.

**Results:**

The overall effectiveness of SKIM was evaluated with 18 targets (14 patients). Direct comparison with FB was performed with 12 targets (10 patients). The vector norm mean ± SD value of motion magnitude under SKIM for 18 targets was 8.2 ± 4.1 mm. The mean ± SD metronome BPM was 54.9 ± 4.0 in this group. The vector norm means ± SD values of target motion for FB and SKIM in the 12 target sub‐group were 14.6 ± 8.5 mm and 9.3 ± 3.7 mm, respectively. The mean ± SD metronome BPM for this sub‐group was 56.3 ± 2.5.

**Conclusion:**

Compared with FB, SKIM can significantly reduce respiratory motion magnitude of thoracic targets. The difference in maximum motion reduction in the overall vector norm, S‐I, and A‐P directions was significant (*p* = 0.033, 0.042, 0.011). Our proposed method can be an excellent practical alternative to conventional compression due to its flexibility and ease of implementation.

## 1 INTRODUCTION

Respiratory motion management has been vital in thoracic or abdominal treatments where tumor motion often significantly and drastically increases the internal gross target volume (iGTV) and introduces motion‐related imaging artifacts.^1^, ^2^, ^3^ Effective motion management is critical to reduce target volumes and improve sparing of critical structures, especially in stereotactic body radiation therapy (SBRT) where hypofractionation is employed.^4^, ^5^, ^6^

For full duty cycle radiotherapy, four‐dimension CT (4DCT) simulation while the patient is free breathing (FB) is the simplest method to complete planning imaging for lung tumors. However, the lack of breathing guidance and significant respiratory motion can result in considerable tumor motion of ≥2.5 cm and large iGTV size.^7^ In order to limit tumor movement, compression has been utilized for breathing motion management for many years.^8^, ^9^, ^10^ Compression force is applied on the abdomen to limit diaphragm excursion, and many articles report its effectiveness in reducing the iGTV while retaining the full efficiency of beam delivery.^11^ However, compression may cause patient discomfort due to the application of a compressing force on the patient's abdomen (e.g., an arch frame with a compression‐plate, a balloon and/or belt, etc.), which may contrarily increase intra‐fraction motion.^10^, ^12^ In many cases oxygen is given to the patient during simulation and treatment adding extra technical difficulties. Furthermore, anatomical deformation taking place under the compressional force can be inconsistent throughout the course of treatment, leading to decreased setup reproducibility.^13^

Several recent studies have developed motion management strategies aimed at improving the reproducibility of breathing and patient compliance during the beam delivery time. Capaldi et al. introduced a mobile app that can provide real‐time respiratory motion.^14^ The respiratory motion data is acquired from an instant respiratory feedback (IRF) system placed in the same location as the real‐time position management (RPM) system to trace a signal simultaneously. Sadeghi et al. developed a capacitive‐sensing monitoring system (CMS) for real‐time monitoring of respiratory motion without contact with the patient and surrogating markers.^15^ The device is comprised of a copper conductive sensor mounted on an acrylic horizontal plate above the patient's chest or abdominal area which detects respiratory motion with high temporal frequency (200 Hz) through the patient body. The system is presented as prototype design and is not yet ready for implementation in the clinic due to requirements to be made of radio‐transmissive material and reduction in size to prevent gantry collision.

Meanwhile, the global spread of SARS‐CoV‐2 (COVID‐19) required major changes to clinical operations throughout healthcare systems.^16^, ^17^, ^18^, ^19^, ^20^, ^21^, ^22^, ^23^ This includes reducing unnecessary contact between staff and patients as well as thorough sanitation of shared equipment, in particular for SBRT treatments that shared motion management aids such as compression devices between patients. Removing the need for such ancillary devices would not only save time and resources but also may increase patient safety under current circumstances.

Our work was initiated to establish a method that is: (1) simple but can derive a similar or improved effectiveness to compression belt while keeping full duty‐cycle and (2) functions without any physical contact with a patient, and finds a way to minimize exposure time during patient contact and setup. In this study, we sought to overcome such drawbacks of traditional motion management strategies by realizing the concept of shallow kinetics induced by a metronome (SKIM). Metronome devices are often used by musicians to reach a constant beat and are readily available for purchase. With SKIM, patients are instructed to breathe at a higher‐than‐normal frequency, and therefore with a reduced breathing magnitude, with the aid of the metronome. Although the volume per breath is smaller than for normal respiration since the breathing rate is increased, it was confirmed with patients that the increased breathing rate was comfortable and they were able to maintain the increased pace at the time of CT simulation. This innovative approach has been implemented in our clinic, and we report our early experience with the first fourteen patients with lung cancer.

## 2 METHODS

The first fourteen lung SBRT patients who were treated under SKIM during the initial COVID‐19 pandemic situation (between April 2020 and August 2021) were selected for inclusion. Patient demographics and treatment site details are summarized in Table 1. Note that beam delivery for patient 1 and 3 were made with the stereotactic technique but not with ablation intention. The retrospective study was part of the protocol reviewed and approved by the medical ethics committee at our institution (No. HM20017385).

**TABLE 1:** Patient demographics in the SKIM study.

Patient #	Sex	Age	Dose (Gy)/fraction	# of fractions	Total dose (Gy)
1	M	55	3	10	30
2	M	58	10	5	50
3	M	58	3	12	36
4	M	59	7.5	8	60
5	F	91	10	5	50
6	F	73	10	5	50
7	M	59	12	4	48
8	F	72	12	4	48
9	M	69	12	4	48
10	M	70	10	5	50
11	M	76	7	5	35
12	F	64	10	5	50
13	M	34	18	3	54
14	F	66	7	5	35
Mean ± SD	F(40%), M(60%)	65.0 ± 12.2	8.6 ± 4.1	6.0 ± 2.6	43.6 ± 12.0

**FIGURE 1:**
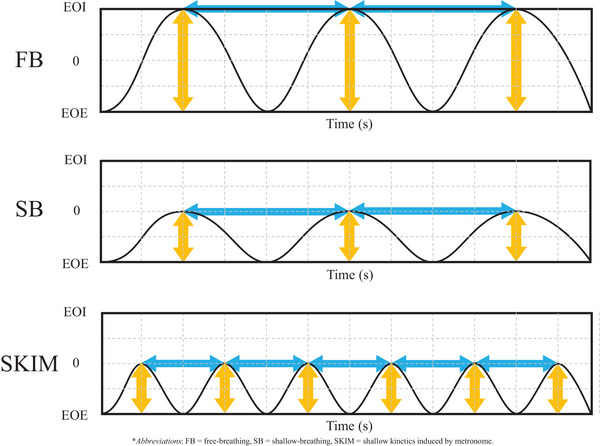
Illustration of breathing patterns, free breathing (FB), shallow breathing (SB) and shallow kinetics induced by metronome (SKIM). Blue arrows indicate the period of breathing cycle while yellow ones do the amount of air inhaled per breathing cycle. *Note*: The total amount of air inhaled in SKIM is same as in FB but breathing magnitude in each breathing cycle in SKIM is half of that in FB in this simple illustration.

**FIGURE 2:**
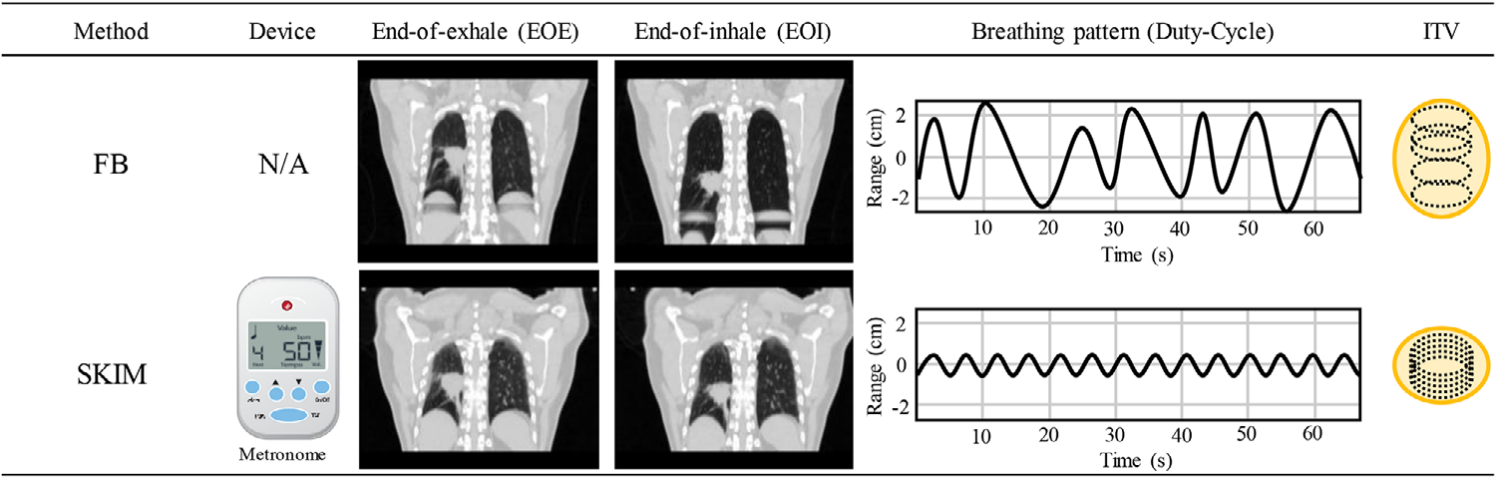
A brief comparison between free‐breathing (FB) and shallow kinetics induced by metronome (SKIM), presents different features, including breathing patterns and internal target volume (iGTV). Our proposed SKIM works with a metronome within a range between 50 and 60 BPM, enabling not only to reduce the breathing motion but also regularize the breathing pattern. *Note*: The breathing patterns and iGTV above are for illustrative purpose only and do not reflect a clinical scenario.

Treatment simulation for SBRT patients included in this study began with the acquisition of a ten‐phase 4DCT for assessment of target motion. When the 4DCT scan with FB showed target motion approximately ≥10 mm a physicist was involved in training the patient to follow SKIM. The target motion assessment is generally completed in the coronal plane and assessed the motion in the superior and inferior direction. The patient was requested to shallowly inhale and exhale in sync with the beats of the metronome with the goal of taking short, shallow breaths rather than attempting to take in their normal volume of air at an accelerated rate. A conceptual illustration of the breathing pattern of SKIM is shown in Figure 1. A new 4DCT was then acquired with the patient under SKIM which was used instead of the FB 4DCT for treatment planning purposes. We utilized a beats‐per‐minute (BPM) range of 50−60 to induce fast shallow‐breathing (FSB), with the exact value selected according to patient comfort and breathing ability. Each dataset was acquired using a spiral pitch factor of 0.1. Usually, the limiting factor are patients that have slow breaths, and the minimal pitch must be used to acquire adequate sampling. Since our patients will breath faster than usual for conventional 4DCT, the pitch was maxed out to 0.1. That pitch is recommended for patients with a breathing rate of 20 breaths per minute. Sixty beats per minute, which converts to 30 breaths per minute was the maximum tempo used and it was the upper limit for most patients to achieve FSB comfortably and for extended period of time. This was the maximum pitch of the Brilliance Big Bore CT scanner (Philips Medical Systems, Cleveland, OH). The metronome used had ±0.001% tempo accuracy and a range of 30 to 280 BPM (M50, Shenzhen Meideal Musical Instruments Co., Ltd). The metronome device is shown in Figure 2 (USD $5 in 2021).

Once the 4DCT images were obtained, the datasets, which contained 10 sets of respiratory phases from 0% to 90%, were exported to MIM software (version 6.9.3, Cleveland, OH) for further processing. Target contouring was completed by a single physician to maintain consistency. The GTV (gross tumor volume) was initially manually delineated on a single respiratory phase. Using the MIM software, the initial GTV volume was transferred to the other respiratory phases using deformable propagation. This propagation was reviewed and corrected, as necessary, in all phases to ensure accuracy. A final iGTV (internal gross tumor volume, simply called iGTV hereafter) was created by combining the GTV volumes from all respiratory phases.

Once target contouring was done the target motion was measured using the contour centroid in 3D (i.e., X = lateral, Y = anterior‐posterior, Z = superior‐inferior directions and vector norm) as defined by the contouring software (MIM Software Inc., Cleveland, OH, USA). The contour centroid was recorded for each phase and the difference was compared to the 0% phase (end of inhalation). The maximum deviation to the 0% phase was recorded in each direction and for the overall vector norm. The mean and standard deviation (SD) values were analyzed for each motion magnitude. A one tailed T‐test for two dependent means was calculated for each direction and the vector norm with a significance level set at *p* < 0.05. The difference between the maximum motion between SKIM and FB for each direction is tested. The maximum motion was defined as the largest distance in each direction between the 0% phase and all other phases. This was patient dependent as some patients the largest difference happened on different phases for each direction. However, most of the time this occurred at the 50% phase. The percent difference between the iGTV with FB and SKIM is calculated. Figure 2 illustrates the major differences between FB and SKIM for one of the patients included in this analysis.

## 3 RESULTS

### 3.1 4DCT data set

Out of 14 patients the full 4DCT data set under SKIM was retrievable for 14 patients and 3 patients among them had multiple targets (2, 2, and 3 targets, respectively). Regarding FB CT data set, however, only 12 FB 4DCT targets were available. Thus, while the overall effectiveness of SKIM was evaluated with 18 targets, direct comparison with FB was made with 12 targets.

### 3.2 Overall effectiveness of SKIM

The vector norm mean ± SD values of motion magnitude of the target for SKIM was 8.2 ± 4.1 mm. Target motions in each direction are summarized in Table 2. The mean ± SD tempo speed for this group was 54.9 ± 4.0 BPM.

**TABLE 2:** The target motion with shallow kinetics induced by a metronome (SKIM) for the 18 targets.

Target #	Patient #	Target location	BPM	SKIM GTV volume 30% phase (cc)	Max deviation from EOI (mm)			Vector norm (mm)
					LAT	A‐P	S‐I	
1	1	RLL	50	81.25	1.02	2.41	15.21	15.43
2	2	LLL	55	5.47	1.04	3.56	13.06	13.53
3	3	RLL	55	57.56	0.26	1.87	7.18	7.36
4	4	RLL	58	44.19	0.5	0.81	5.9	5.94
5	5	LLL	55	3.88	0.9	1.7	6.5	6.74
6	6	LUL	50	1.56	0.64	0.28	0.36	0.73
7	7	RLL	52	0.83	2.3	4.89	12.32	13.45
8	8	RML	58	1.41	1.51	0.73	3.08	3.38
9	8	LLL	58	1.47	2.01	0.77	6.6	6.94
10	9	RUL	58	1.82	0.4	0.6	3.9	3.95
11	10	RUL_sup	45	0.74	1.2	0.79	7.9	7.93
12	10	RUL_inf	45	38.21	1.38	2.03	4.22	4.7
13	11	LLL	58	118.77	1.8	2.7	8.8	9.11
14	12	RLL	58	4.28	0.9	3.1	14.1	14.45
15	13	RLL	58	2.86	2.7	1.2	12	12.2
16	13	LUL	58	1.08	2.19	0.96	3.95	4.55
17	13	RUL	58	0.99	4.89	1.1	5.73	7.49
18	14	RUL	58	9.46	1.2	2.3	10	10.27
	Mean ± SD		54.9 ± 4.0	20.9 ± 33.1				8.2 ± 4.1

*Note*: Directions are LAT = lateral, A‐P = anterior‐posterior, and S‐I = superior‐inferior. All units for motion are in mm. The max deviation from end of inhalation (EOI) in each direction may occur during different phases. The vector norm is only reported for the largest calculated difference between a single phase and EOI. Note the vector norm deviation is a linear distance and often slightly shorter than the actual curved trajectory (i.e., hysteresis).

### 3.3 Comparison between FB and SKIM

**FIGURE 3:**
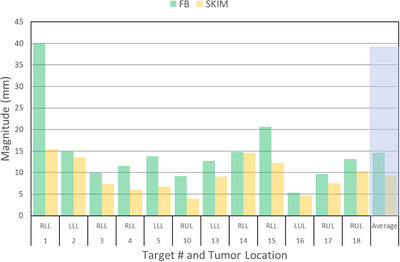
The comparison of the vector of motion magnitude of the target between free‐breathing (FB) and shallow kinetics induced by metronome (SKIM) for the 12 targets. The vector mean ± SD values of motion magnitude of the target for FB and SKIM were 14.6 ± 8.5 mm and 9.3 ± 3.7 mm. *Note*: Both FB and SKIM data sets were retrievable for 12 targets only (out of 18 targets) thus, comparison was limited to them.

The vector norm mean ± SD values of motion magnitude of the target for FB and SKIM were 14.6 ± 8.5 mm and 9.3 ± 3.7 mm, respectively with an average decrease of 5.4 ± 4.7 mm. Target motions differences and difference in iGTV between SKIM and FB are summarized in Table 3. SKIM showed a target motion reduction in most cases, including up to 24.6 mm improvement in vector norm for patient 1. Among the cases with SKIM in this 12 target group, the mean ± SD tempo (metronome) speed was 56.3 ± 2.6 BPM. Figure 3 compares vector norm target motion between FB and SKIM for the 12 targets for further illustration. The one tailed T‐test showed that the difference in maximum motion reduction in the overall vector norm, S‐I, and A‐P directions was significant (*p* = 0.033, 0.042, 0.011, respectively). The maximum motion reduction in the lateral direction was not significant (*p* = 0.29385). The iGTV is reduced for all targets, except for a single site which shows a small increase (less than 5%). The average percent decrease of the iGTV volume is 20%.

**TABLE 3:** Comparison of the target motion between free‐breathing (FB) and shallow kinetics induced by a metronome (SKIM).

					Max deviation from EOI (mm)					
Target #	Patient #	Target location	Breathing	BPM	LAT	A‐P	S‐I	Vector (mm)	Diff^a^ (mm)	iGTV percent Diff^b^
1	1	RLL	FB		1.53	1.72	40	40.03	−24.6	−36.4
			SKIM	50	1.02	2.41	15.21	15.43		
2	2	LLL	FB		1.08	3.65	14.41	14.87	−1.34	2.7
			SKIM	55	1.04	3.56	13.06	13.53		
3	3	RLL	FB		0.71	3.72	9.22	9.94	−2.58	−5.1
			SKIM	55	0.26	1.87	7.18	7.36		
4	4	RLL	FB		1.22	3.1	11	11.49	−5.55	−24.7
			SKIM	58	0.5	0.81	5.9	5.94		
5	5	LLL	FB		4.4	4.3	12.5	13.78	−7.04	−45.1
			SKIM	55	0.9	1.7	6.5	6.74		
10	9	RUL	FB		1.5	1.4	8.9	9.13	−5.19	−29.2
			SKIM	58	0.4	0.6	3.9	3.95		
13	11	LLL	FB		1.1	2.1	12.6	12.71	−3.6	−6.8
			SKIM	58	1.8	2.7	8.8	9.11		
14	12	RLL	FB		0.5	2.5	14.8	14.84	−0.39	−21.4
			SKIM	58	0.9	3.1	14.1	14.45		
15	13	RLL	FB		2.5	3.15	20.3	20.62	−8.42	−10.6
			SKIM	58	2.7	1.2	12	12.2		
16	13	LUL	FB		0.79	1.59	5.08	5.38	−0.83	−21.4
			SKIM	58	2.19	0.96	3.95	4.55		
17	13	RUL	FB		5.75	3.9	6.78	9.71	−2.22	−19.0
			SKIM	58	4.89	1.1	5.73	7.49		
18	14	RUL	FB		0.7	2.9	12.95	13.2	−2.93	−20.8
			SKIM	58	1.2	2.3	10	10.27		
	Mean ± SD			56.3 ± 2.5			FB	14.6 ± 8.5	−5.4 ± 4.7	−19.8 ± 12.9
							SKIM	9.3 ± 3.7		

*Notes*: Directions are LAT = lateral, A‐P = anterior‐posterior, and S‐I = superior‐inferior. All units for motion are in mm. The max deviation from end of inhalation (EOI) in each direction may occur during different phases. The vector norm is only reported for the largest calculated difference between a single phase and EOI. Note the vector norm deviation is a linear distance and often slightly shorter than the actual curved trajectory (i.e., hysteresis).

^a^ Diff = SKIM‐FB.

^b^ Percent Diff = 100(SKIM/FB – 1).

## 4 DISCUSSION

The principle of SKIM is straightforward, speed up breathing frequency while reducing the breathing amplitude to limit tumor motion. The increased breathing rate is assessed by medical physicists with the patient to ensure the patient can comply through the 4DCT and during treatment. It is an intuitive methodology and adaptable to the patient's breathing ability, with a range of breathing rates that provide reduction.

**FIGURE 4:**
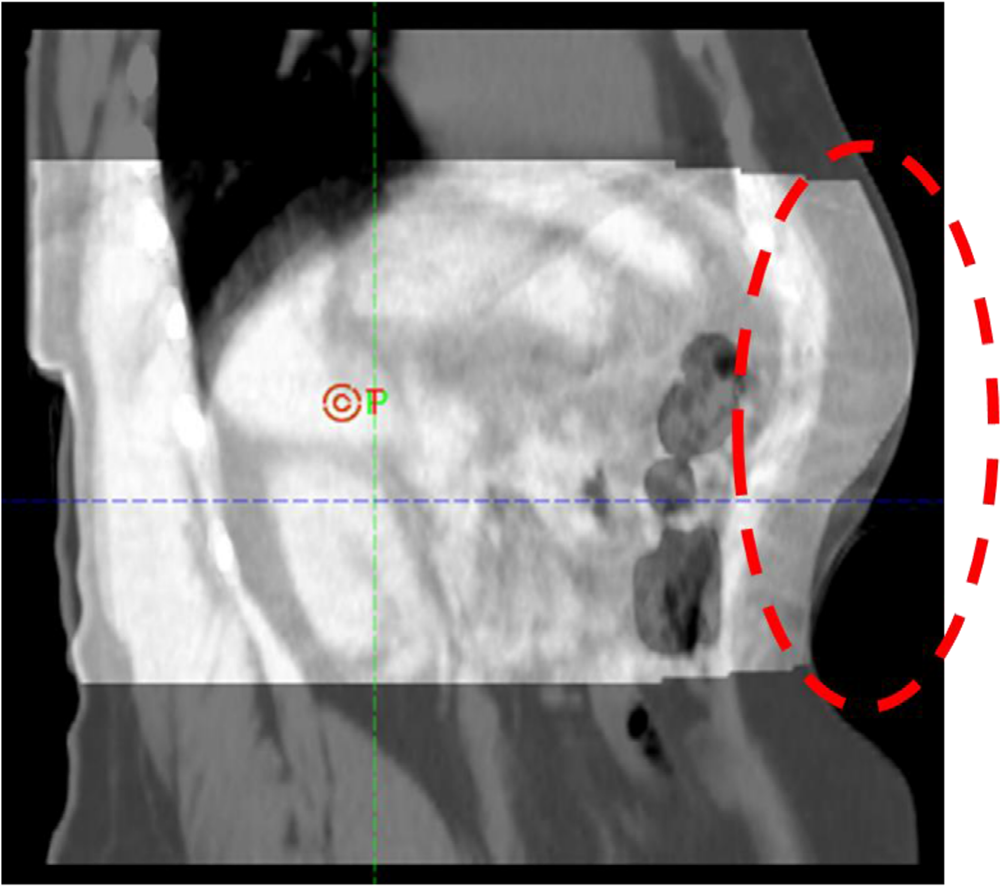
Example of difference in the external body contour with hard compression between CT simulation and treatment CBCT.

There are several compression devices either in rigid (e.g., arch frame + plate) or non‐rigid (e.g., balloon or belt) versions. Regardless of rigidity, compression devices may deform the anatomical structures during treatment sessions, reducing reproducibility. This can commonly occur when the exact placement of compression is variable between CT simulation and treatment. An example of the difference in the body extent due to variable compression is evident in Figure 4. These devices are not universally sized for all patients either. Compared to abdominal compression, SKIM is contactless and not subject to distortion. However, SKIM requires active patient participation to achieve voluntary motion management. When it comes to inducing SKIM, the range of 50−60 BPM setting seems quite beneficial to gain the meaningful advantage of shallow breathing while respecting patient comfort. Here, the BPM corresponds to twice the respiratory cycles per minute (CPM) or respiratory rate; that is, 30 CPM matches 60 BPM. In general, there is a limitation of breathing speed when scanning a patient with 4DCT. Thus, it is essential to check the specific capabilities and limitations of the CT scanner.^24^, ^25^, ^26^ For example, the maximum value of BPM for the Brilliance Big Bore CT is 60.

Generally, the application of physical abdominal pressure to minimize a motion magnitude is widely adopted in the clinic. Heinzerling et al. observed a motion reduction of both lung and liver lesions with the application of high compression.^27^ The mean gain of motion reduction of the target compared between no compression and high compression was 6.4 mm. Eccles et al. also studied the effectiveness of the compression method in reducing target motion amplitude with a reported mean gain from the abdominal compression of 2.3 mm in the SI direction for liver tumors.^28^ From our study the vector norm mean for the target motion reduction comparing between FB and SKIM was 5.4 mm for the target, showing at least the equivalent of using conventional compression methods. However, further studies are required to obtain concrete results using a systematic comparison of both physical compression and SKIM in the same population. When comparing the percent difference in iGTV volume between FB and SKIM, there was a considerable reduction in size for all patients except patient 2 for whom iGTV was 2.7% larger with SKIM than that of FB. Two explanations, may be possible. First situation was that the actual curved trajectory (i.e., hysteresis) of tumor motion was longer with SKIM compared to FB while the straight vector norm motion was larger. The other case was that breathing pattern in FB scan was irregular and generated artifacts, causing missing parts of tumor in some phases while SKIM less artifact prone due to well‐regulated breathing pattern. Such artifacts are often clearly observed in coronal images as illustrated in Figure 2 (see the area of liver dome). Considering that SKIM showed smaller motions in all 3 directions (see Table 3) we believe the latter was the more convincing scenario. Obviously, causing less artifacts is another advantage of SKIM since more reliable delineation can be achieved.

Use of abdominal compression has another limitation identified by Bouilhol et al. who reported that compression impact on tumor motion depended on the location of the tumor, with upper and middle lobe tumors showing minimal to no benefit from compression and potential for increased motion.^10^ This was theorized to be due to changes in patient anatomy and increased use of upper thoracic region in breathing motion induced by compression. There does appear to be some improvement in upper lobe targets with reduction in the range of 5.2 to 0.8 mm which is within the overall range of our population. As the SKIM method does not alter patient anatomy, this limitation was eliminated using our method. Alternatives to compression for motion management have been previously implemented.^29^, ^30^, ^31^ The Active Breathing Coordinator (ABC), for instance, had been widely used because it enabled active enforcement of breath‐hold at the desired lung volume, requiring a nose clip and mouthpiece which is single‐use. In general, the ABC device is challenging to use for patients, especially those with atypical mouth anatomy or other conditions that make the use of the mouth or nose pieces difficult. The current pandemic poses new and serious problems for the ABC device.^20^, ^21^, ^32^ While it is known to be safe in terms of influenza and upper respiratory infections (URIs) due to filtration within the air channel, it is not clear how patient safety may be affected with respect to COVID‐19. Based on current information from the product suppliers, the single‐use mouthpiece/tube with the Viromax filter has 99.99% filtration efficiency for viral particles of 0.1 μm in size.^19^ However, the device manufacturers do not have data on filtration efficiency for smaller particle sizes than 0.1 μm. While influenza is much larger than 0.1 μm, COVID‐19 size is cited to be 0.06 to 0.14 μm in diameter.^33^, ^34^ Furthermore, every breath‐hold technique suffers from significantly deteriorated duty‐cycle, causing noticeable increase of contact and care time. Therefore, in the face of such uncertainty, our clinic has sought alternative methods.

SKIM requires more patient participation compared with compression, while still being less invasive than ABC. This is one of the primary drawbacks of SKIM. If the patient is not willing or able to follow the instructions, SKIM may not be an appropriate fit. However, the vast majority of patients are willing to adhere to instructions during treatment. In our experience with the initial cohort of patients, we found improved patient performance and comfort by clearly explaining the goal of SKIM technique to take short, shallow, smooth breaths. Once a clear and concise training method was developed with the first few patients, the coaching time was minimal for subsequent patient simulations (∼ 5 min). In our clinic, the coaching is similar to DIBH coaching in that the process and importance of the patient participation and repeatability was stressed. The patient then practiced breathing using the highest BPM that was tolerable. The patient was watched throughout the practice session that occurs prior to the CT scan. During that time the patient was accessed for adherence by watching the anterior skin of the torso. This was assessed over thirty to forty‐five second intervals to ensure compliance could be achieved throughout a CT scan and treatment. Once adherence and repeatability was confirmed, the CT scan was completed. Occasionally, repeating the coaching process was necessary at the beginning of the first treatment fraction as the patient did not remember the breathing instructions given during their simulation a week or 2 priors. Again, the retraining took only a few minutes. Overall, coaching patients requires a brief but clear training session that adds a negligible amount of time to both simulations as well as treatment delivery. In addition, patients are encouraged to exercise SKIM at home under a similar condition as treatment using either a physical or virtual metronome, with instructions given for finding smartphone or computer metronome applications. The current study focused on SBRT patients that had 4DCT for both FB and SKIM, however SKIM can be used for other treatments beyond hypo‐fractionation in which patient motion is a concern without adding extra time required for other motion management devices. During patient treatments, the video monitoring systems in the console area can be utilized to monitor the consistency of shallow‐breathing patterns with the set BPM speed from the metronome. The machine was not configured for gating, so the beam was controlled manually if patient was no longer compliant. The verification was largely qualitative and was used to ensure that the breathing period did not vary after in‐room coaching during image and treatment.

As with most conventional IGRT, the daily CBCT is relied upon to ensure the patient is setup correctly but is also used to ensure that the extent of the tumor motion matched well with the planned iGTV and PTV. The surface tracking was used as an additional layer to verify the breathing speed of the patient during the initial setup, imaging of the patient, and delivery. The surface imaging ensured that the period remained consistent during imaging to produce the compression effect was as effective as in the CT simulation. Once the CBCT was completed, the alignment is the same as for conventional IGRT, using the iGTV and PTV to align the tumor for daily treatment. Then breathing speed is continuously monitored through beam delivery except when visibility is lost due to camera blockage at certain gantry angles.

Although no such case was experienced during this study, if the motion on the CBCT exceeds what was planned for the patient, they may need to be re‐simulated. In the case of SKIM, it could be that the patient was not adhering to the metronome and in which case more training is provided and the CBCT should be repeated. The cohort used here was relatively small and every patient was in good compliance, it is possible that some patients may not be able to repeat the BPM used during CT (probably due to deteriorated condition) and would require a re‐simulation with a different motion management technique (most likely compression). Additional studies using real‐time imaging techniques such as kV x‐ray with implanted markers and in‐room MR may be useful to further investigate the inter‐treatment variability and the sensitivity of optical surface monitoring with SKIM. While surface imaging can be useful it is not mandatory for the implementation of SKIM. Typical in‐room lasers and patient monitoring cameras can also be used instead of a surface imaging system.

**FIGURE 5:**
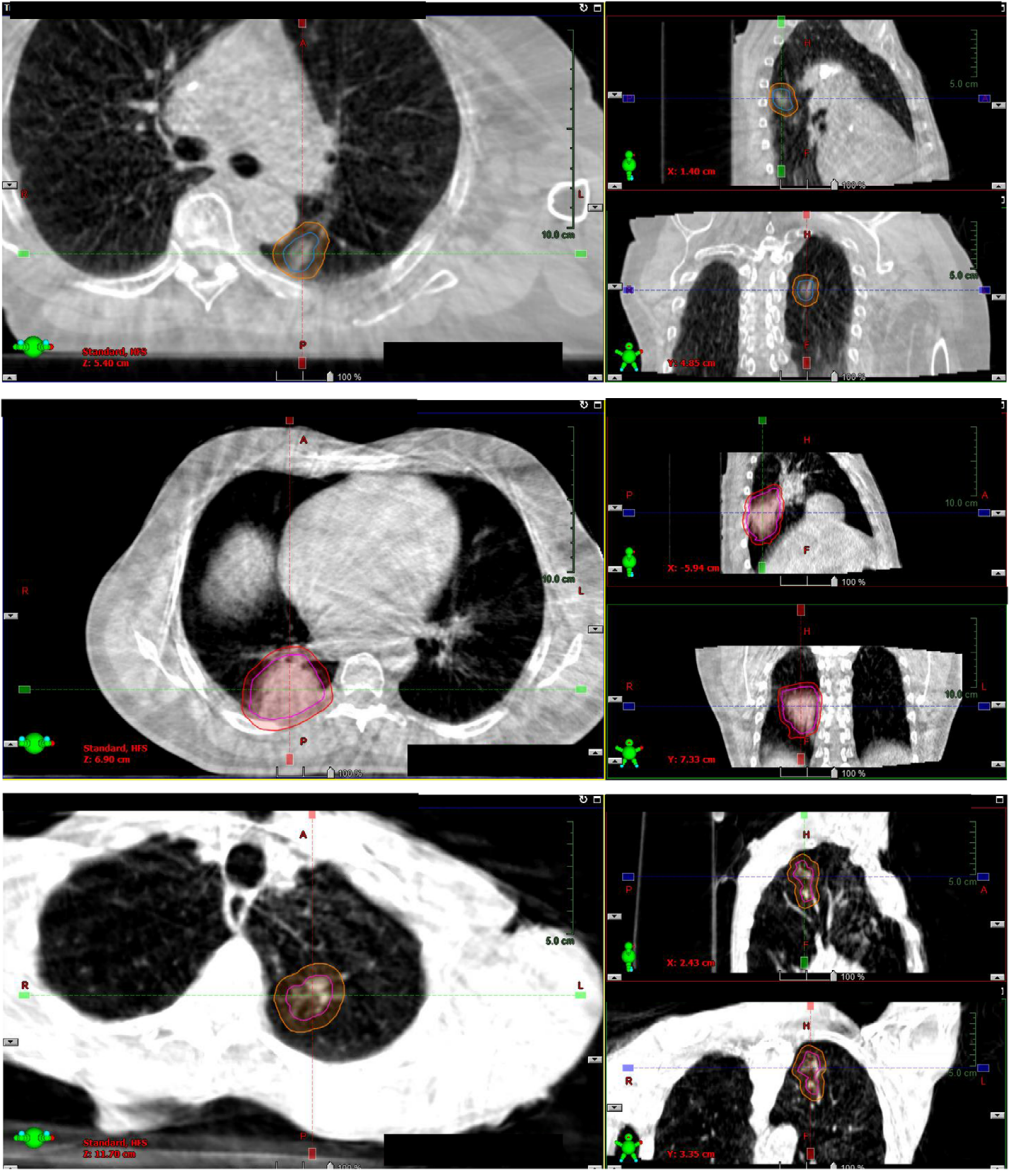
SKIM CBCT alignment with the iGTV and PTV displayed based on the CBCT alignment for patient 8 (LLL target), patient 1, and patient 6. These cases represent a moderate, high, and low degree of tumor motion, respectively.

While not part of this study, as mentioned above, the daily CBCT was used to ensure the patient's anatomy matched well with the planning CT. This is inherently an additional validation of the SKIM methodology, as the tumor motion would be different if the metronome beat was not followed. This CBCT was verified by physician, physicist, and therapists prior to treating. Alignment was done to match the iGTV, PTV, and normal structures as best as possible. Three examples of CBCT alignment compared to planning image are shown in Figure 5.

Besides the numerous available online applications, the price of a physical metronome is not a significant barrier for implementing SKIM, as these can be found on the internet market for less than $10 as of today (year 2023). SKIM also does not require sterilization of equipment and saves setup time for both CT simulation and treatment compared to compression and ABC. Therefore, we believe our approach, can be easily implemented by any clinic throughout the world regardless of departmental budget and resources available to the radiation therapy program even after the COVID‐19 pandemic is over.

## 5 CONCLUSION

SKIM is a simple and easy‐to‐setup strategy for respiratory motion management that does not require complicated and/or uncomfortable setup devices. We have demonstrated that SKIM can significantly reduce the respiratory motion magnitude in SBRT cases compared with FB, with no additional motion management apparatus. This provides a significant advantage compared to other motion management strategies, especially under the COVID‐19 pandemic and beyond. Our proposed method can be an excellent practical alternative to any forced compression or forced breath hold methods due to its flexibility and ease of implementation.

## AUTHOR CONTRIBUTIONS

James Sohn contributed to study design, data collection, analysis, and writing of the manuscript. Mitchell Polizzi contributed to data collection, analysis, and writing of the manuscript. Reed McDonagh contributed to data collection, analysis, and writing of the manuscript. Christopher Guy contributed to data collection, study design, supervision of the project, and writing of the manuscript. Rabten Datsang contributed to data collection, study design, supervision of the project, and writing of the manuscript. Elisabeth Weiss contributed to study design, supervision of the project, and writing of the manuscript. Siyong Kim contributed to study initiation, overall supervision of the project, and writing the manuscript. All authors discussed the results and contributed to the final manuscript.

## CONFLICT OF INTEREST STATEMENT

The authors declare no conflicts of interest.

## INSTITUTIONAL REVIEW BOARD APPROVAL

The retrospective study was part of the protocol reviewed and approved by the medical ethics committee at our institution (No. HM20017385).

## Data Availability

Research data are stored in an institutional repository and will be shared upon request to the corresponding author.
